# Young adults’ views on the mechanisms underpinning the impact of pets on symptoms of anxiety and depression

**DOI:** 10.3389/fpsyt.2024.1355317

**Published:** 2024-02-15

**Authors:** Roxanne D. Hawkins, Chih-Hsin Kuo, Charlotte Robinson

**Affiliations:** ^1^ Clinical and Health Psychology, School of Health in Social Science, The University of Edinburgh, Edinburgh, United Kingdom; ^2^ Psychology Division, The University of Stirling, Stirling, United Kingdom; ^3^ School of Psychology, Cardiff University, Cardiff, United Kingdom

**Keywords:** anxiety, coping, depression, human-animal interactions, pets, self-harm, suicide, wellbeing

## Abstract

**Introduction:**

Emerging adulthood is considered a peak age for the onset of mental health difficulties with approximately 75% of mental health disorders being diagnosed during this developmental period. Companion animals confer both risk and benefits to mental health yet the potential underpinning mechanisms which explain such impacts are not fully understood. This study aimed to gather an in-depth understanding of young adults’ lived experience of how their companion dogs and cats may impact their mental health symptoms and the perceived mechanisms which explain their effects.

**Methods:**

Semi-structured interviews were carried out with 16 young adults aged 18-26 years, from the United Kingdom, who either had a companion dog, cat, or both. All participants had difficulties with anxiety and or depression, and 12 had received a formal diagnosis of an affective disorder.

**Results:**

Five overarching themes and one subtheme were identified through reflexive thematic analysis using an inductive approach: Theme 1: Pet impact on generalized anxiety and panic, Subtheme 1A: Pet impact on social anxiety and loneliness; Theme 2: Pet impact on low mood, depression, and stress; Theme 3: Pet impact on severe mental health and suicide prevention; Theme 4: Staying well; Theme 5: Positive outlook and successful futures. Several perceived mechanisms underpinning the impacts of pets for mental health were also identified.

**Discussion:**

These findings have relevance for the development and evaluation of mental health interventions and treatment protocols aimed at young adults with mental health difficulties, where companion animals may prove to be effective for symptom management and improvements in positive wellbeing.

## 1 Introduction

Mental health problems remain one of the main causes of overall disease burden worldwide and represent the largest single cause of disability in the United Kingdom (1, 2). There is an increased risk for psychological vulnerability in emerging adulthood, typically defined as the ages between 18 and 29 years (3, 4). Emerging adulthood spans a key developmental age where there is a transition from late adolescence into adulthood whereby societal, psychosocial, and biological factors can increase stress and cause psychological distress (5). For example, changing social roles and identities, increased risk-taking, and heightened instability are distinctive features of this period that can increase negative emotions and lower wellbeing. The availability of social support and family bonds is critical for navigating through the difficulties and uncertainties of this life stage (6, 7). Statistics have shown that the 12-month prevalence of any psychiatric disorder, particularly anxiety and mood disorders, is more than 40% in people aged 18–29 years (8) and that approximately 75% of mental health disorders are diagnosed by the end of this developmental period (9). The problems that emerge in young adulthood can persist long-term over the life course (3), yet there tends to be low help-seeking within this age group (10), and many young adults within the UK do not have access to appropriate mental health support (11). Moreover, suicide is a leading cause of death for young people under the age of 35 years in the UK (12), so identifying preventative and protective factors for young people’s mental health is a key public health concern.

The preventive effects of companion animals, hereby referred to as “pets”, for mental health difficulties have been a topic of interest in recent years, within both the public media and scientific investigation. With the rise of “pet therapy” (animals being utilized in therapeutic work usually involving interaction between an individual, pet handler, certified animal, and healthcare professional; e.g., 13) and the Mental Health Foundation, a UK-based charity, now recommending pets as a source of improving mental health (14), it is important to disentangle the impacts of pets for psychological health. Quantitative studies have yielded mixed and inconclusive findings, and longer-term impacts of pets remain unclear due to the reliance on cross-sectional and correlational designs, as well as variability in measures meaning difficulties with comparison and replication (15–17). Existing studies have also been critiqued for overlooking the complexity and individualities of each human–pet relationship through attempts to identify a relationship between pet ownership and decreased mental health symptoms (e.g., 18, 19), whereas the same pet could both exacerbate and reduce mental health symptoms depending on the context and type of interaction (20). It may be the case that pets help to manage mental health symptoms, offering a temporary sense of relief and preventing the worsening of symptoms, rather than eliminating them. Few studies have investigated the impact of pets on those with clinical levels of mental health difficulties, and even fewer have investigated the impact of pets on mental health within emerging adulthood when relationships with pets can offer a stable source of comfort and support in times of uncertainty and instability.

Young adults facing adversity place high importance on their pet relationships and view them as key mental health supports, helping to manage mental health symptoms and enabling them to effectively deal with major life stress (21). In the same study, young adults reported that their pets promoted positive self-image and boosted their confidence. Increased positive self-regard and related wellbeing factors were also found for pet-owning marginalized (e.g., LGBT+) emerging adults in the USA who are at increased risk for vulnerability due to adversity, discrimination, and social disparities (22, 23). Another study found that pets were featured within young adults’ discourse of wellbeing, with the value being placed on meaningful interactions with pets (24). For emerging adults within the USA, dealing with hardship and avoidance of loneliness were key motivations for pet ownership (25), and research supports the impacts of pets on reduced loneliness and social anxiety within emerging adulthood (26). Although emerging adulthood is a period of increased independence, exploration, and freedom, pets can offer an important sense of autonomy, responsibility, structure, and stability, all of which are important contributors to emotional health (27). It is important to note here that negative impacts of pets on mental health have also been identified. For example, in research with adults, pets were reported to exacerbate mental health difficulties including increased maladaptive guilt, stress, worry, and caregiver burden (20); such rumination and worry can increase the risk for affective disorders (28). Furthermore, many adult pet owners report housing and financial concerns, which could be exacerbated in emerging adulthood due to a lack of stability and financial security in this life period (27). Both potential risks and benefits of pets for mental health should, therefore, be considered.

Given the increased vulnerability to mental health difficulties within emerging adulthood and the proposed preventative effects of pets for mental health symptoms, the psychological implications of human–pet interactions may be particularly pertinent during this developmental stage. However, as mentioned, few studies have explicitly investigated the impact of pets on the mental health of young adults with anxiety and depression, and emerging adults within the UK remain an underrepresented population within this field of work. Quantitative research does not allow for the exploration of the nuances of human–pet relationships, such as individual pet effects (and pet type) and the mechanisms that explain both the benefits and risks of pets for mental health. Qualitative lived experience research offers the opportunity to explore such mechanisms within this developmental period, thus understanding the why and how behind the impact of pets on mental health. The current study, therefore, aimed to gather an in-depth understanding of young adults’ lived experience of how their companion dogs and cats may alleviate or exacerbate their mental health symptoms. This project focuses on affective disorders (anxiety and depression), the most prevalent mental health difficulties in this population (9). Dogs and cats were chosen because they are the most common pet types within the UK and are most often talked about in relation to mental health (20, 29).

## 2 Materials and method

### 2.1 Design and participants

A qualitative approach was undertaken whereby in-depth semi-structured interviews were carried out online. Inclusion criteria included the following: 1) aged 18–29 years; 2) had a pet dog, cat, or both; 3) lived in the United Kingdom; 4) fluent in the English language; and 5) struggling with anxiety and/or depression/low mood. A clinical mental health diagnosis was not a requirement for participation due to low help-seeking within this population (10).

Our sample (N = 16) included 14 individuals who self-identified as female and two participants who self-identified as non-binary. The age range was 18–26 years (M = 22). Most participants (n = 11) identified as heterosexual, five identified as LGBTQ+, and one answered “other”. Most participants lived in England (n = 14), one participant lived in Scotland, and one lived in Ireland. Most participants were in a romantic relationship (n = 11) and living with their caregivers (n = 7). Six participants had a pet cat, six participants had a pet dog, and four participants mentioned having both. Most participants (n = 12) lived with their pets at the time of the study, and most (n = 13) did not have any other types of pets. Length of pet ownership ranged from 5 months to 10 years. Four participants mentioned that their motivation to acquire their pet was for their mental health. Full participant details can be found in **Table 1**.

**Table 1:** Participant demographics.

ID	Pseudonym	Age	Gender	Mental health diagnosis	Pet	Identified difficulties and other relevant diagnoses	Ethnicity	Sexual orientation	Romantic relationship	Accommodation	Living with pet
001	Brynn	23	NB	Y	Dog	Anxiety/social anxiety, depression. Autistic.	Asian/Asian British (Chinese)	Bisexual	Y 5+ years	Homeowner (living alone)	Y
002	Lola	18	F	N	Both	Anxiety/social anxiety, depression.	White*	Heterosexual	Y 3 years	With parents/caregivers	Y
003	Avery	25	F	Y	Dog	Anxiety, hospitalized for mental health. EUPD.	White	Heterosexual	N	With parents/caregivers	Y
004	Isla	20	F	Y	Cat	Anxiety, OCD, previous suicidal thoughts, and depression.	White	Heterosexual	N	With parents/caregivers	Y
005	Ava	25	F	Y	Cat	Anxiety/social anxiety, panic attacks, depression. Suspected BPD, EUPD.	White	Heterosexual	Y 3 months	Shared housing	N
006	Leith	22	F	Y	Both	Anxiety, depression, psychosis (hallucinations), depersonalization, suicide attempts, hospitalized for mental health. BPD.	White	Heterosexual	Y 3 years	With parents/caregivers	Y
007	Osla	19	F	N	Both	Anxiety, trembling, low mood.	White	Heterosexual	Y 18 months	With parents/caregivers	N
008	Rhona	26	F	Y	Both	Anxiety, depression, panic attacks, low mood, and suicidal thoughts in past.	White	Bisexual	Y 2.5 years	Private rented place (cohabiting)	Y
009	Blair	21	F	Y	Dog	Anxiety, panic attacks, depression, low mood, hospitalized for mental health. Autistic.	White	Gay or lesbian	N	With parents/caregivers	Y
010	Skye	25	F	Y	Dog	Anxiety. ADHD. Autistic.	White	Heterosexual	Y 6 years	Homeowner (cohabiting)	Y
011	Aria	25	F	N	Cat	Anxiety/social anxiety, depression, low mood, paranoid thoughts.	Asian/Asian British (Chinese)	Heterosexual	Y 7 years	Private rented place (cohabiting)	Y
012	Edina	24	F	N	Dog	Social anxiety, sadness, thoughts of self-harm. Neurodiverse.	Asian/Asian British (Indian)	Heterosexual	Y 6 months	Shared housing	Y
013	Daisy	19	F	Y	Cat	Anxiety, panic attacks, and depression.	White	Bisexual	N	Student accommodation	N
014	Shona	21	F	Y	Dog	Anxiety, depression, hospitalized for mental health, eating disorder.	Mixed/Multiple ethnic group**	Heterosexual	N	Shared housing	N
015	Erwina	25	F	Y	Cat	Anxiety, panic attacks, low mood, depression. Specific phobia about feeling sick and being sick.	White	Bisexual	Y 5 years	With parents/caregivers	Y
016	Harper	19	NB	Y	Cat	Anxiety, mood swings.	White	Other	N	Student accommodation	Y

NB, non-binary; OCD, obsessive-compulsive disorder; BPD, borderline personality disorder; EUPD, emotionally unstable personality disorder; ADHD, attention-deficit hyperactivity disorder.

*Scottish/English/Welsh/Northern Irish/British.

**Any other mixed/multiple ethnic background. Note that private rented place (cohabiting) meant living with a romantic partner.

Despite the previously reported low help-seeking in our population, 12 participants in our sample had received a clinical diagnosis for anxiety, depression, or both, and 13 participants reported having sought help for their mental health, predominantly counselling, therapy (e.g., cognitive behavior therapy), or medication. Some participants mentioned previously having suicidal thoughts or having been hospitalized due to their mental health. Additional diagnoses, co-diagnoses, and difficulties had also been identified including personality disorders, eating disorders, attention deficit disorder, obsessive-compulsive disorder, psychosis, and paranoia (see **Table 1**). Three participants were autistic, and one other participant reported being neurodivergent.

### 2.2 Procedure

Purposive and convenience sampling was used whereby participants were recruited *via* an advert on the UK MQ Mental Health research platform (n = 6) or through an advert on social media channels including Twitter and Facebook (n = 10). Recruitment ended when the target sample size (n = 16) was achieved. This sample size was deemed adequate for achieving theoretical saturation in qualitative designs (30). Interested participants were able to scan a QR code or access a web link to an online sign-up survey. The sign-up survey provided detailed information regarding the study and its procedure so that participants were able to provide full consent. Once participants had read the information sheet, they were directed to the next page, which was an online consent form. Participants who provided consent were then taken to a short demographic survey. Following this, participants completed an online form to indicate their time preference for the interview and were informed that they would be contacted by the researcher if they met the inclusion criteria.

All interviews were 1:1 with a single experienced researcher and took place online (Microsoft Teams, n = 15) or by telephone (n = 1). At the beginning of the interview, participants were reminded of their rights and the study’s aims and purpose and were able to ask questions about the study. Given the sensitive nature of the topic, a document containing a list of mental health resources was provided by email along with health and behavior support resources for pets. An emergency contact for the interviewee was also requested prior to the interview commencing, and the researcher monitored the participant’s mental health throughout the interview. If participants had both dogs and cats as pets, they were able to talk about both within the interview but were asked to make it clear which individual pet they were referring to in their answers. The interviews were audio recorded for transcription purposes. The interviews lasted between 16 and 37 minutes, with an average length of 23 minutes. At the end of the interview, participants were again able to ask any questions regarding the study before being emailed a de-brief form that thanked them for their participation and provided them with more information regarding the study with some additional relevant resources. Participants were also sent a £20 shopping voucher as a thank-you for taking part.

Demographic questions included the following: age, gender identity, ethnicity, sexual orientation, relationship status, type of accommodation (e.g., homeowner and student accommodation), and presence of children. Participants were then asked the following questions about their mental health: 1) Have you been struggling with anxiety, depression, or both? 2) Have you ever had a formal mental health diagnosis? 3) Have you ever sought professional help for your mental health? 4) Do you experience any other mental health difficulties? Participants were able to provide further details if they wished. Participants were then asked several questions about their pet including: 1) type of pet (whether a cat or dog or both); 2) number of cats/dogs; 3) whether currently living with their pet; and 4) number and type of any other pets owned. Interview questions were built around the human–pet relationship, e.g., “What does having a pet mean to you?”, and pet impact on mental health, e.g., “Do you think your dog/cat has had an impact on your overall health and wellbeing?”, “Do you think your dog/cat has had any impact on your feelings of anxiety (if relevant)?”, and “Do you think your dog/cat has had any impact on your mood or depression (if relevant)?”. Prompts for each question were used, e.g., “If yes/no, in what ways? Has your dog/cat helped with specific symptoms?”

### 2.3 Data analysis

Reflexive thematic analysis (TA) was used to analyse the data. TA involves a six-step coding process that includes disassembling and reassembling data and searching for patterns and meaning within the data, with the overall goal of finding overarching themes and subthemes. Although the interview questions asked about the impact of pets on mental health specifically, the questions were kept broad, and a flexible, inductive, and data-driven approach was undertaken (31, 32). A collaborative approach was undertaken whereby all researchers were involved in the coding process and agreed on the final themes. Our sample size was deemed more than sufficient for this type of data analysis (33, p. 50).

## 3 Results

Five overarching themes and one subtheme were identified through reflexive TA: Theme 1: Pet impact on generalized anxiety and panic, Subtheme 1A: Pet impact on social anxiety and loneliness, Theme 2: Pet impact on low mood, depression, and stress, Theme 3: Pet impact on severe mental health and suicide prevention, Theme 4: Staying well, and Subtheme 5: Positive outlook and successful futures. Several mechanisms underpinning the benefits of pets for mental health were identified through data analysis, and these are presented in **Table 2**. Real names have been replaced with pseudonyms throughout the results.

**Table 2:** Identified mechanisms underpinning the benefits of pets for anxiety and depression.

Mechanisms underpinning benefits of pets for mental health		
Pet behavior: proximity seeking, eye contact, “watching” or “checking” behavior, attentiveness, and responsiveness, attuned to emotions, physically affectionate, sleeping in proximity, sounds (e.g., purring)	Human–pet behavior: physical touch/petting, observing pet, taking photos, looking at photos of pet, talking to/confiding in pet, including pets in social interactions and activities, talking about pet to others, eating at set mealtimes with pet, shared activities, and quality time together	Pet perceived as consistent, readily available, as enjoying time spent with owner, perceived learning from them
Increased mindfulness, focus on the present moment	Increased sense of safety, protection, and reassurance, reduction/prevention of harmful thoughts	Company and comfort without judgement, expectations, or pressure. No opinions or negative feelings towards owner
Distraction, modify attention away from worries, rumination, and paranoid thoughts	Identification with pets and sense of mutual support	Increased joy and pleasure, comic relief, and sense of fun
Increased relaxation and sense of calm	Social catalyst/facilitating social interactions	Feeling valued and appreciated
Feeling loved or “chosen”	Help with disengaging from social interactions	Increased sense of purpose. Feeling needed and/or relied on for care
Better emotion regulation	Increased energy and encouragement to engage in healthy activities	Increased company/companionship
Increased physical exercise and time spent in nature	Increased motivation/will to live	Reminder to “keep tabs” on own mental health and to engage in self-care
Increased sense of responsibility and caregiving role	Increased sense of routine and structure	Increased self-control, sense of independence
Feeling productive, a sense of achievement and self-pride	Increased resilience and ability to cope with stress, adversity, and severe trauma, and stress relief	Sense of emotional support
Motivation to seek mental health treatment and to avoid re-admission to hospital	Increased optimism, hope, and positive outlook	Motivation to continue/pursue work and/or education

### 3.1 Theme 1: Pet impact on generalized anxiety and panic

For generalized anxiety problems (symptoms reported by most young adults in this study), pets reduced symptoms by helping to promote a sense of mindfulness, allowing young people to focus on the present moment, thus acting as a distraction away from worries. This is described by Rhona:

“*They’re just so in the moment and you know they’re not thinking about life worries. They’re just thinking about what’s happening then and I think you can learn a lot from animals to just kind of appreciate the moment*” (Rhona).

A pet’s ability to modify attention away from rumination and negative thinking patterns was also described by Skye, who had difficulties with intrusive paranoid thoughts:

“*I have a lot of paranoid thoughts about people. Like all the time, and having my cats around, serves as a distraction, because she’s always doing something interesting and then I’ll be distracted and look at her and like, try to take photos of her*” (Skye).

A commonly perceived benefit of pets was the reduction of physiological symptoms of anxiety such as trembling hands, as well as the de-escalation of panic attacks. The mechanisms underpinning these effects appeared to be physical touch and petting, feelings of comfort, proximity, and the regulation of breathing, as described by participants Rhona, Ava, and Blair:

“*That feeling of comfort can just kind of reduce the feeling of panic you know, the anxiety might still be there very much mentally and physically, but it can reduce it, can stop it getting worse to a certain extent, if that makes sense, because, you know, you’re not necessarily alone. You have another wee creature there that’s with you and loves you*” (Rhona).

“*I’ve had a few, like panic attacks. It definitely helps sort of calm me down in those situations, like giving them a stroke or something has quite helped me sort of get a hold of my breathing and stuff*” (Ava).

“*When I had problems about like going to school and going to college, feeling really anxious about those and having panic attacks, I think they, you know, it’s something straight away that could calm you down, especially when you’re in that high kind of emotional state when you’re really panicked*” (Blair).

The word “*calming*” in reference to pets was commonly reported, with physical affection and touch being important mechanisms, which underpinned this effect, along with the pets’ emotional and behavioral state being reflected onto themselves:

“*If they’re calm around me, then there’s nothing to sort of be anxious about. So, when they’re calm, it sort of reflects on to me*” (Lola).

Ava described how having her cat sleeping and purring next to her, as well as petting her cat, helped her to feel relaxed and lowered her anxiety. Pets were, therefore, perceived to have the ability to help regulate negative emotions when in a highly aroused state; this not only alleviated anxious feelings but also helped to increase positive mood and stabilize mood swings:

“*I deal a lot with like mood swings, so erm, he helps me feel a lot more stable. Just having like a stable source of comfort in a way*” (Harper).

This ability to regulate mood was particularly important for one participant who was going through treatment for suspected borderline personality disorder, helping to keep their mood “*steady and stabilized*”. For specific anxiety problems, pets reduce symptoms by increasing a sense of safety. For example, Avery described how her dog helped her to feel safe when home alone, and Lola described how her cat helped with her fear of the dark:

“*I feel like when he is by my side or with me in the same house, I feel a lot less scared I suppose. I am scared without him*” (Avery).

“*I’ve always had a fear of the dark from some childhood problems, and she’d always come in and she would always sleep behind me. So, I knew I was never alone when it came to the nighttime*” (Lola).

Interestingly, identifying with a pet that was also perceived to be anxious helped to promote the human–pet bond and provided a sense of mutual support and anxiety relief:

“*I think it kind of helps in a way that that she can be quite an anxious dog. So it’s kinda like I’m there for her and she’s also there for me. A bit of a mutual support going on. I’m kind of her emotional support human*” (Brynn).

“*I think we’re both anxious in regard to not wanting to be on our own and I think being together helps us feel less alone kind of thing*” (Avery).

### 3.2 Subtheme 1A: Pet impact on social anxiety and loneliness

Social anxiety was a common problem reported, and pets were perceived to be beneficial in two key ways. The first was during social interactions, acting as a social catalyst, increasing social connections, or politely disengaging from social interaction when feeling overwhelmed. The second mechanism was through providing company when socially withdrawing or when feeling lonely or alone. A pet’s ability to be an important “ice breaker” and “social catalyst” was described by Ava and Brynn:

“*Everyone likes talking about their own pets, so like it definitely is like an icebreaker in social situations*” (Ava).

“*She’s a small, cute dog. Everyone’s like, aww, can I say hi to the dog. So I think there’s like a somewhat of a social element as well*” (Brynn).

These social benefits were mostly reported by dog-owning participants; this was because dog owners were able to engage in more social interactions that involved their animals outside of their home, for example, including them in social activities, such as meeting their friends in dog-friendly pubs. When social interactions became overwhelming, pets were a good way to “*disengage*”, and this was particularly important for neurodiverse young people, as described by Brynn:

“*When I’m feeling stressed in a social situation, she very much helps with the kind of almost like a way out. You know, a way of kind of being able to politely disengage and then kind of take a moment to like, regulate*” (Brynn).

Pets also provided non-judgemental company including physical affection “*without pressure*” of verbal communication and without expectations from the social interaction:

“*Social interactions can be a bit much when you’re feeling low, it’s not too much when you have an animal because there’s no expectation there … you don’t have to talk to them, they don’t have to talk back*” (Rhona).

“*Sometimes you know if you have a person next to you when you’re feeling anxious or panicky or whatever they might be trying to speak to you or do something. Sometimes it can make it worse, but with an animal you know, they’re never gonna speak to you or kind of bug you or whatever. They’re just chilling there, and especially coz cats are so, like chill*” (Erwina).

“*Having a pet is like having a friend at home like where he/she wouldn’t really like have opinions or like urgh negative feelings towards me. And I don’t need to overthink if my cat likes me or not like, like it urgh, different from me interacting with people*” (Aria).

Having “*someone there*” or being “*not alone*” was important, especially when socially withdrawing from others, during relationship breakdowns, when “*feeling down*” or “*feeling low*”, or when feeling isolated and experiencing loneliness. Harper talked about living alone and how their pet provided important company and support, especially when they were not feeling well. Consistency, feeling that their pet was readily available, along with the pet being non-judgemental, was also important for many young people:

“*I struggle with like friendships and feeling lonely. So like when she wants to do things with me, it just makes me feel like less alone. She’s just always there and she doesn’t judge me*” (Isla).

“*I think a big part of having mental illness is like loneliness and not having anybody around and sometimes, you know, you just feel so isolated. So it’s nice to, even if you don’t get out and see people you know, like you’re too ill or unwell or whatever, you still have your pet in the house with you. They’re still around like, they’re always there*” (Erwina).

Lola talked about being an only child and how her cats provided company especially when her mum was unavailable or when she felt that she could not confide in her mum during difficult times:

“*Whenever I feel alone or like, I just can’t talk to my mom about things, I’ll just go and spend more time with them*” (Lola).

Edina, talked about her dog being an important source of affection and company following negative social interactions such as arguments or fights with friends or family members. Edina also alluded to the ability of her dog to “check” on her following these negative interactions, seemingly being attuned to her emotions:

“*I usually end up going to my room [after an argument] and then I’ll usually leave the door open, like a little bit, because sometimes [dog] will walk in and she’ll just sit on the bed and like, you know, watch TV with me. And it’s just like, you know, instantly, I just feel better. There’s like another person in the room with me who like isn’t gonna say anything. I know she understands that. You know that what happened like was like a little difficult for me. So she just came to see. It’s like coming to check on me*” (Edina).

The perceived ability of pets to be attuned and responsive to a young person’s emotions, and their “checking behavior”, was also seemingly important for regulating and improving mood and thus reducing symptoms of depression, as reported in Theme 2.

### 3.3 Theme 2: Pet impact on low mood, depression, and stress

Most young adults reported having difficulties with low mood or “*feeling down*”, and some mentioned having difficulties specifically with depression. In relation to their pets, these participants used words such as “*cheer up*”, “*dopamine boost*”, “*mood booster*”, or “*mood lifter*”. One way through which pets improved mood was through laughter and comic relief, with young people reporting that their pets were “*cute*” and did “*silly things*” to make them laugh. Overall, pets increased feelings of joy and a sense of pleasure:

“*We’re there just having a good time and it just really makes me feel like happy and just makes you look at life in a really nice way, just sort of like that I’m just happy with everything and it changes my whole mood, definitely*” (Osla).

“*Every time I saw them, it was like, I felt like ten times better. And I was like, OK, so life is fine. Like, you know, I’m all fine. Everything will be OK and yeah*” (Edina).

Edina continued to describe how even just looking at pictures of their dog when apart helped to lift her mood on difficult days. Pets enable young people to feel valued and appreciated, through the perception that the pet enjoys spending time with them:

“*I think it makes me feel more like I’m valued and like someone appreciates me being there … It makes me feel really happy because it makes me feel like he wants to be around me*” (Harper).

Pets, therefore, helped young people to “*feel better*” and seemingly provided a more positive outlook on life in general, even without daily physical contact. Important mechanisms through which pets improved low mood were through providing company, as well as being attentive, responsive, and physically affectionate:

“*I mean the feeling depressed and sad as well I think coz again, it’s just that company like I might just be sitting on my bed feeling like crap or whatever and urm. He’s just around. He’s there. It’s nice to have, like, another living thing around with you*” (Avery).

“*She has this way of being incredibly persistent in trying to cheer you up. It’s very difficult to continue crying and being upset when a dog with an almost sandpaper tongue won’t stop licking your face. So that, I mean, certainly you know, it’s highly effective*” (Bryn).

For low mood and depression, an important reported benefit of pets was an increased sense of purpose. Young people reported that their pets helped them to “*get up*” and “*out of bed*” and to engage in healthy activities such as going for a walk outside:

“*You know, some days I wouldn’t be able to even get out of bed and you know I couldn’t do anything. Now I get up every day for him. You know, I get up every day. I want to get up for him and take him outside and you know go for walks and play with him. You know it’s really really made an impact*” (Leith).

This sense of purpose was facilitated through a sense of responsibility and caretaking role within the pet’s life and feeling “*needed*”:

“*Before, I sort of feel like I didn’t really have much of a purpose, I suppose, but I think him showing that, you know he loves me, and he needs me. And you know that has got such a positive impact on me that you know I need to get up every day for him you know*” (Leith).

For Rhona, this sense of purpose was important following a traumatic event in her life, which left her struggling with her mental health:

“*About five years ago, I did have quite a traumatic event that happened in my life and just everything changed. I was taken out of university and stuff like that, and I was at home and that’s when I got the dog, and she just give me such a purpose. It’s always give me a sense of purpose and if my mental health was ever at a very low point, I could look at them and remember that I’m there for them*” (Rhona).

This sense of purpose helped young people to stay well, which is expanded upon in Theme 4, as well as being important for suicide and self-harm prevention as described in Theme 3.

### 3.4 Theme 3: Pet impact on severe mental health and suicide prevention

Most young adults reported severe mental health difficulties, with mentions of previous self-harm and being hospitalized due to suicide attempts. Additional diagnoses were also reported including eating disorders, psychosis, obsessive-compulsive disorder, and specific phobias (as reported under the subtheme for anxiety). For these participants, additional benefits of pets were reported that are worth mentioning here. For example, Shona described how eating at set mealtimes with her dog was helpful:

“*I’ve struggled with an eating disorder. It’s silly in a way to think about. But like the fact that he [dog] will eat at set times, I’d just sit and eat with him. So, I guess in that way like it was nice to just have the company*” (Shona).

One participant, Leith, who experienced psychosis, mentioned that although she still hallucinated, she felt that these hallucinations had reduced since having her pet dog. Leith described how her dog was responsive and seemed to sense when she was having symptoms; her dog sought physical affection and proximity, which helped to reduce symptoms:

“*I used to get like a lot of hallucinations and things which obviously would give me a lot of anxiety. I think of like situations in my head that make me anxious and things. And you know, I think he [dog] can, he can sense it, you know, because once I get into that sense he comes and sits on me or sits next to me and just lays his head on me and sitting there just stroking him. It all, it all just goes away, you know? And it’s crazy to think that an animal can do that really, he really, really can*” (Leith).

Pets were also talked about in relation to suicide prevention, being an important “*protective factor*” and helping to prevent “*dark thoughts*”, with strong beliefs about a pet’s ability to prevent harmful thoughts and acts of future suicide attempts:

“*I used to get really dark thoughts, and in 2021, you know I had quite a major suicide attempt. I was in hospital for quite a long time, and again I’ve noticed since that, I’ve had the dog, I haven’t thought that way at all. I was only thinking this the other day I was like I don’t remember the last time I felt that way and it genuinely has been since I’ve got my dog*” (Leith).

One participant, Shona, spoke about her dog being helpful once discharged from an inpatient unit, helping her to keep safe, especially during periods of solitude such as when her mum was away:

“*…But because my dog was there like, he helped keep me safe in a way. So I had something to do, like to look after, and it gave me sort of like a focus and the motivation to keep on like*” (Shona).

Along with this sense of focus and motivation, participants reported that pets prevented thoughts and acts of suicide through a sense of connectedness and bond to the animal and not wanting to “*leave*” them. Pets also provided a sense of “*hope*” and a “*reason to live*” through feeling needed and responsible for their pets’ care:

“*I do remember feeling quite suicidal at one point and looking at her and being like I can’t leave her. And she was such a protective factor for me. In that time she was just such a wee ray of sunshine that I didn’t have anywhere else in my life at that time*” (Rhona).

“*He [dog] would come and visit with my parents when I’d be allowed outside the ward or whatever. And I think that helped mental health, cause it was kind of like something to keep hopeful for. And you know, when I was at my lowest and thinking about things, it was something that was like oh, you know like a reason to live and stuff I guess*” (Blair).

“*But like they’re a responsibility. So like when I was depressed like, I would have suicidal thoughts and like, it would go through my mind that like, but I need to take care of them*” (Isla).

Beliefs surrounding a pet’s ability to understand and respond to human feelings were also reported upon in relation to suicide prevention. For example, one participant, Edina, had strong beliefs surrounding her dog’s ability to pick up on her thoughts of self-harm, seeking proximity and eye contact with her in a perceived attempt to prevent her from harm, as well as feeling as if there was someone “*watching*” and protecting her:

“*…I feel like they somehow knew [they were having harmful thoughts], but they will just come up and like, check on me and like they just come and sit with me, sometimes they would both sleep on my bed. It was almost as if, like, there was like another person in my room … sometimes they’re, like, just glared at me when I was talking something really stupid … Like there’s been days when I have been really sad, I’ve had like thoughts of self-harm. Sometimes I’ve had thoughts and like you know, the dogs have been around, and it was sort of like another person who’s watching me have these thoughts and I just. I just felt like ‘ohh what am I doing’ like you know or like, you know things are hard and they will get better*” (Edina).

It is important to note that for one participant, Blair, losing her family pet that she was strongly attached to increased harmful thoughts of suicide, and this was her first experience of bereavement:

“*Losing him was I guess very impactful for me. And I guess the, you know, the suicidality and the thoughts and things like that, that was very strong at that time because attachment was lost*” (Blair).

### 3.5 Theme 4: Staying well

This theme describes young adults’ beliefs about the ability of their pets to help them “*stay mentally healthy*” through “*keeping tabs*” on their mental health and to foster increased self-control and motivation to engage in activities or behaviors that will maintain positive wellbeing and reduce negative symptoms. Part of this was their pet’s reliance on them for their care and needs to be met:

“*You kind of have to, have that like control over yourself when something is relying on you. Quite a protective factor in terms of keeping somewhat at least keeping tabs on my own health. You’re kind of always reminded like, you know, I have to kind of stay well, I guess*” (Brynn).

“*The fact that like, he needs things, and like he needed me. Urm he got me out of bed, like I take him for a walk, I have things I need to do for him*” (Shona).

A common reflection was that pets helped to improve wellbeing and were a motivator to “*keep well*” by providing routine and structure. Through caring responsibilities, pets helped to increase a sense of independence, especially when living alone, which in turn increased wellbeing through increased motivation to look after themselves. A sense of purpose, feeling productive, self-pride, and a sense of achievement were also commonly referred to by pets in helping to keep mentally healthy:

“*It gives me that sort of sense of like purpose, you know? Like if I wasn’t here, you know, I feel like they would be upset and I, you know, I don’t want, don’t want that for them*” (Leith).

“*I was really sick and just like, really depressed, and I barely went to school. When I got him, because I was at home like all the time, it was suddenly just like this, this living thing that I could take care of. It was something that almost, you know, like felt productive. It was like an achievement, you know*” (Erwina).

Part of keeping well was increased motivation and encouragement to “*get up and do things*”, to go outside for a walk or to play, increasing overall energy and time spent in nature and engagement in physical activity. Therefore, this mechanism was mostly relevant to dog owners:

“*I know that going outside objectively is good for me, but oh God, do I not want to do that? That’s like the last thing I want to do … so I think having a dog in that sense like definitely it means that I’m doing that on a regular basis*” (Brynn).

“*…and I’ve sort of been inside all day, like feeling like a bit like unmotivated and then the dog was sort of like, want to go out for walks and stuff. It helps me to get outside and do things as well and that also improves my mental health*” (Osla).

“*…that’s [dog walking] the most physical activity that I get sometimes. So it’s really helpful with, like when I have like severe anxiety, going out and getting that fresh air really helps*” (Edina).

It is worth noting here that young people were asked a question in the interview regarding potential pet impact on healthcare decisions relating to mental health, but most young adults reported no impact. However, quite a few participants had received medical treatment for their mental health, and pets were reported to have increased their motivation to stay well and avoid going back to the hospital or increased their motivation to seek mental health treatment:

“*I’ve been in hospital with my mental health five times. Erm, and he’s [dog] been (one of my reasons) to get like, get well and get out of hospital kind of thing*” (Avery).

“*I remember thinking I need to get myself sorted … I did probably seek therapy if I was, you know, looking at my dog and thinking that she was only reason I was there*” (Rhona).

“*I was in hospital for a little while, partly due to my mental health. I was there for a bit. I think part of erm, sort of wanting to get me home was my cat. Erm well, the home environments’ generally better obviously. But like I think part of sort of wanting to get home to my cat and stuff and it being more helpful to have them*” (Daisy).

One participant spoke about how having her cat present and near to her during online counselling sessions improved the sessions through decreasing anxiety and increased reassurance and sense of safety:

“*I’ve seen a few different counsellors with regards to my depression and my anxiety, but I thought I’d never really got anywhere with them. A few of the meetings I had with them was during Covid when I was at home, and I felt when I talked to them, I sometimes will get anxious in myself. So when I spoke to them at home having like I said, one of my cats next to me just gives me that reassuring feeling that I am at home. I am with my cats in a safe place*” (Lola).

### 3.6 Subtheme 5: Positive outlook and successful futures

This theme encapsulated beliefs about pets having a positive life impact through increasing resilience and ability to cope with daily stressors as well as adversity and stressful life periods. Pets also increased optimism and enabled a more positive outlook on life, increasing chances of successful futures. Many young adults talked about how their pets helped them to cope with university stress, especially around exam times and assignment deadlines, being important sources of emotional support, helping to reduce stress, and being a welcome distraction and sense of relief, which in turn helped them to stay in university and to complete their academic work:

“*I’m in my last year of university now, but erm, I’ve had to take interruptions and so many extensions and things like that just because I couldn’t cope with it. And since I’ve now got him, I’ve come back to university, I’ve handed in assignments on time, and I found it altogether less stressful*” (Leith).

“*I think during that time [exams] my partner’s dog like, really helped and just sort of being with them, spending time with them, just taking my mind off exams … like the day that I had erm sort of finished my exams, I went to see my partner and his dog as well, and it was just nice to have that sense of relief, and also seeing the dog as well and just experiencing that happiness with them, almost like sharing it with them in a way*” (Osla).

Being a “*consistent*” source of support, spending time together, and increasing a sense of mindfulness or distraction away from worry and stress were important for completing assignments and for coping with multiple life stressors and worry about university:

“*Like I’ve had different stresses over the years, you know, like work and that kind of thing. Urm and she’s [dog] always, she’s always there. She’s a consistent help*” (Skye).

“*I’d come back from like classes and I just sit with her all day. And she’d sit with me, even while I was doing assignments and she generally just keep, like, you know, made me feel better*” (Edina).

“*A lot of my problems that I’m thinking about, like work stresses, kind of disappear or kind of my mind is taken off and just kind of focused on him [dog]*” (Blair).

A pet’s ability to understand and respond to human emotions as well as their “*checking*” behavior were also important for stress relief during difficult life periods and academic study:

“*There’s been times where I’ve been, like, quite like heightened stress, and he [dog] definitely picks up on it and you know, like, comes over and like, sort of like checks if you’re OK*” (Shona).

“*When they do wanna cuddle up with me, I actually get that sense of calm. Coz college at the minute, it’s been very stressful. I’ve been very stressed. So, when I go home, it’s a nice relief to see them there and that they’re happy to then come and sit with me*” (Lola).

Skye spoke about how her cat motivated her to continue with her studies as the qualification would help her to find a job and ultimately provide financially for her cat:

“*Having my cat around gave me a reason to keep going with the course like that, because I need to take care of the cat. And you know, I need to think about the future as well and so somehow I would want to be responsible and like, try to complete the course and (stuff), not dropping out and urm like a giving the cat away*” (Skye).

For one of the neurodiverse young adults, Edina, who had difficulties with school due to severe social anxiety, her dog was an important stress reliever and helped to increase her coping ability and provided a more positive outlook:

“*I was generally always anxious that I you know, gonna have like a mental breakdown, but then every time I come back from classes and then I would take the dogs for a walk, and it would just feel better by itself. It was sort of like the dogs were giving me some like, you know, internal like release of like stress that I was like, OK, you know, I’m walking these dogs and like, you know life can’t get better than this. I don’t really have to worry about uni, it’s gonna work out. And it was just like, you know, really help me feel better*” (Edina).

Lastly, there were quite strong beliefs about pets’ ability to positively change their lives and enable positive futures. One young adult in particular, Leith, spoke in detail about her dog making her “*a different person*”, helping her to pursue both work and education, providing a sense of hope and optimism about the future:

“*I think I just really just really want to emphasize, you know, especially with my dog, you know? Because I feel like I can’t emphasize it enough the difference he’s made. Like if I look back at the person I was, you know, last year before I got him, it’s a completely different person to who I am now. I’ve managed to now get a job you know, as well as doing university and hopefully graduating in September and you know, I look back like last year and I used to think these things weren’t gonna be possible for me. And so it’s just, you know, it’s just amazing. The difference he has made to me*” (Leith).

## 4 Discussion

This study has provided an in-depth understanding of young adults’ lived experience of how their companion animals impact their symptoms of anxiety and depression. Several perceived underpinning mechanisms (which span across several outcomes/themes) explaining such effects were identified and could be tested within future quantitative studies. First, it is important to acknowledge that the young adults in our study reported quite severe mental health difficulties despite having their pets yet also displayed strong beliefs surrounding the beneficial impact of their animals for the reduction of symptom severity and the management of symptoms. Strong language was used around the perceived impact of pets, such as “always”, “definitely”, “massive”, “absolutely”, and “amazing for mental health”. However, it was mentioned that perhaps pets provided temporary relief from psychological distress, such as providing symptom relief in the moment of pet interaction, rather than long-term symptom prevention, possibly explaining the inconclusive evidence found in quantitative correlational studies (17, 34). Despite this potential temporary sense of mental health impact, several important perceived benefits in relation to pets and mental health were reported that are noteworthy.

Generalized anxiety symptoms were commonly reported by the young adults in our study, and pets were perceived to reduce such symptoms in several key ways: first, through physiological pathways such as regulating breathing and emotion regulation and lowering physiological arousal (e.g., through touch and petting), helping young adults to feel calmer and more relaxed (e.g., through physical affection), which helped to de-escalate panic attacks, in line with previously proposed biological and physiological benefits of pets (35, 36) and therapy animals (37). The findings relating to pets’ ability to help regulate negative emotions, stabilize mood swings, and increase positive mood are in line with developmental studies with children (38) and adolescents (39). Through such calming effects, pets also help to increase mindfulness and the ability to be “present” and “in the moment”, which is important for anxiety management (40). Pets also modify attention away from worry and rumination, negative thinking patterns, and harmful thoughts, all of which contribute to the maintenance of anxiety and depression (41, 42). Such harmful thoughts often included self-harm and suicide, and young adults viewed their pets as an important source of attachment and protection (e.g., through a pet’s “watching” behavior and proximity), providing a sense of safety, security, and reassurance, preventing harmful thoughts and actions, and thus preventing future admissions to hospital or inpatient units. This sense of safety and security was also important for anxiety and fear management. A pet’s ability to prevent harmful thoughts was further facilitated through an increased sense of hope, focus, and motivation to “carry on” and through providing a “reason to live”. Our findings, therefore, demonstrate the important role that animals can play in suicide prevention and animals’ role in keeping young adults safe, supporting previous research with both neurotypical and neurodiverse adults (43–45). However, one participant mentioned increased suicidal thoughts following pet bereavement, so further support is needed for young adults experiencing the loss of a pet, such as the development of effective coping mechanisms (46).

Social anxiety and reported loneliness were common among our young adult sample, and pets, particularly dogs, were perceived to provide social benefits through facilitating social interactions (e.g., including pets within social interactions) and through promoting favorable social support relationships and feelings of social connectedness, supporting previous evidence of the “social catalyst” effects of pets that can bolster mental health (47, 48). However, social withdrawal was also commonly reported due to low mood, and pets enabled young adults to politely disengage from social interaction when they were feeling overwhelmed; this was particularly important for neurodiverse participants. During social withdrawal and isolation, both dogs and cats provided important companionship, being readily available when young adults felt too unwell to socialize; thus, pets prevented feelings of isolation and loneliness (although we note that research on the impact of pets on loneliness in adult samples has been inconclusive; 49). Although talking to and confiding in pets have been identified as an important source of support in previous studies, particularly in the absence of human social support (50, 51), for our sample of emerging adults, it was important to receive unconditional and non-judgemental company and affection from pets, without the pressure to verbally communicate, such as being asked to talk about their feelings, thus demonstrating the unique social and emotional benefits of pets for mental health, compared to human companions.

The young adults in our study felt that both pet dogs and cats were attuned and responsive to their emotions, and this was important for improving low mood; such sensitivity and responsiveness can aid attachment development and have been found to be important for mental health in previous studies with adults (52, 53). Consistency, predictability, proximity, eye contact, and shared meaningful activities (e.g., play) and mutual enjoyment (e.g., enthusiasm during human–pet reunion) are also important for human–pet bonding, particularly for dog owners (52), and were important mechanisms identified by the young adults in our study that explain the mood-enhancing effects of pets. The “mood-boosting” effects of pets were also attributed to the ability of both dogs and cats to increase fun and laughter, thus reducing symptoms of anxiety and depression through comic relief, in line with past human–animal interaction (HAI) (20, 54) and mental health research (55). Feeling loved, cared for, and valued by a pet was also important for young adults, and these mechanisms increased positive emotions and optimism and enabled a more positive outlook, thus further demonstrating pet impact on hedonic and eudaimonic wellbeing (56). Previous research has demonstrated that pets can increase resilience during times of adversity (57), and our study supports this, as young adults reported that their pets aided their ability to cope with worry and daily life stressors as well as more stressful life periods including trauma. This increased coping ability and resilience meant that young adults were more likely to persist with academic studies, which in turn meant more successful and positive futures for them, a novel HAI finding that our study has highlighted.

Our study provides further evidence for the importance of routine and structure for the management of mental health symptoms in young adults that can be provided through pet care; such responsibilities increase a sense of purpose, supporting previous HAI findings with family research and older adults (58, 59). This sense of responsibility facilitated behavioral activation, encouraging young adults to engage in healthy activities such as physical exercise and time in nature, thus enabling them to stay well. It should be noted, however, that physical exercise was not as prominent a theme in our emerging adult data as it has been in other studies with older adults and family studies (60, 61). This may be attributed to the inclusion of companion cats in our study or to the living situations of our sample whereby many young adults lived with parents, meaning potential shared responsibilities such as dog walking. The young adults in our study also reported that through taking care of a pet’s needs, they felt more able to keep tabs on their own mental health, acting as a motivator or reminder for self-care and to stay well themselves. Taking care of a pet also increases a sense of independence, self-pride, and achievement, supporting past research with child and adolescent samples (62). These are, therefore, further unique ways in which pet care responsibilities can aid positive mental health.

It is important to consider that those young adults who signed up for the study may have done so because of their strong attachment to and beliefs regarding the positive impacts of pets, as reflected in their personal accounts. This meant that perhaps we were unable to capture weaker bonds and more negative impacts and risks to pets’ wellbeing. This limitation has been reflected in the field in general, along with the bias of predominantly female participants, also reflected in our sample. Our study was limited to the UK and participants were predominantly White British. Our sample did include however non-binary and LGBT+ individuals and those who are neurodiverse and underrepresented groups within the HAI field. Future studies should aim to examine both risks and benefits of pets for mental health across more diverse samples, particularly for neurodiverse young adults given the unique findings found in our study (e.g., social disengagement) and previous studies (63, 64). Our study purposively recruited individuals who had difficulties with anxiety and/or depressive symptoms, and most of the sample had a clinical diagnosis of an affective disorder. We were, therefore, able to examine the impact of pets on those with clinical levels of mental health difficulties. Co-diagnoses were common, and so our study offered some insight into other underrepresented mental health difficulties within the field (e.g., borderline personality disorders, eating disorders, and psychosis). However, such insight was limited, as questions were not designed to probe further information given the focus on anxiety and depression, and so further investigation into the role of pets in the management of symptoms for more severe mental health difficulties will be important. Interestingly, our study found that when anxious participants viewed their pets as also being anxious, they found comfort in their perceived mutual understanding and shared identity with the pet. It would, therefore, be interesting to investigate in future whether anthropomorphism (attributing human emotions to animals) plays a role in human and pet health, as well as whether emotions can transfer between owners and pets (e.g., see 65, 66).

A strength of our study is that we examined both dog- and cat-owning young adults, addressing the lack of data regarding mental health and cat ownership (67). Human–cat interactions and activities can differ from those with dogs, and so mental health benefits could also differ. However, most mechanisms identified by young adults in our study (except for social activities and physical activity) applied to both dogs and cats with similar positive impacts being reported. Both dogs and cats could, therefore, be incorporated into mental health self-care plans that aim to manage and reduce symptoms of anxiety and depression. Although out of the scope of the current paper, individual pet temperaments were important to young adults. For example, an energetic dog provided mental health benefits for some, but for others, a quieter, calmer, and more affectionate dog was preferred. Therefore, mental health benefits may depend on additional factors that should be further examined in future studies such as owner preferences (e.g., for certain breeds, personalities, or temperaments). Additional factors could include attitudes (68), perceived compatibility (69), relationship quality (70), specific types of pet activities and interactions (38, 56, 67), and the presence or absence of pet behavioral and health problems (71); these may be more important for mental health than simply comparing pet types (e.g., dogs *vs.* cats). It should be noted that this study was qualitative in nature, so the impacts of pets and potential underlying mechanisms explaining such effects are based on young adults’ views and personal experiences, and causation cannot be determined. It is, therefore, recommended that future large-scale quantitative studies further test the mechanisms identified by the young adults in our study in relation to mental health outcomes.

## 5 Conclusion

This study found that young adults in the UK perceive dogs and cats to have positive impacts on symptoms of anxiety and depression by providing temporary symptom relief in the moment of pet interaction. Specific mutual activities (e.g., playing and walking), physical affection (e.g., petting), pet availability, attunement, responsiveness, pet behavior (e.g., proximity seeking and eye contact), and social and psychological factors (e.g., sense of safety, positive affect, and mindfulness) provided important symptom relief, were important for keeping well, and played a role in the prevention of self-harm and suicide. The underlying mechanisms (e.g., coping, motivation, and positive outlook) also played a role in positive futures for young adults such as increasing perseverance in academic studies. Longer-term impacts of pets remain unclear, paving the way for more longitudinal designs that incorporate mixed methods for triangulation of data. These findings have relevance for the development and evaluation of mental health interventions and treatment protocols aimed at young adults with mental health difficulties, where companion animals may prove to be effective for symptom management and improvements in positive wellbeing. Ethical issues and the welfare of companion animals should be considered within such mental health care plans. It is also important to consider that pet interaction will not always lead to positive benefits, and individual circumstances (e.g., commitment, expectations, resources, and finances) should be evaluated prior to pet acquisition.

## Data availability statement

The datasets presented in this article are not readily available because of participant privacy and ethical considerations. Requests to access the datasets should be directed to roxanne.hawkins@ed.ac.uk.

## Ethics statement

The studies involving humans were approved by Clinical Psychology, University of Edinburgh Ethics Committee. The studies were conducted in accordance with the local legislation and institutional requirements. The participants provided their written informed consent to participate in this study. Written informed consent was obtained from the individual(s) for the publication of any potentially identifiable images or data included in this article.

## Author contributions

RH: Conceptualization, Formal analysis, Funding acquisition, Investigation, Methodology, Project administration, Supervision, Writing – original draft, Writing – review & editing. CK: Formal analysis, Writing – review & editing. CR: Data curation, Formal analysis, Investigation, Methodology, Writing – review & editing.
